# Baseline [^18^F]FDG PET/CT and MRI first-order breast tumor features do not improve pathological complete response prediction to neoadjuvant chemotherapy

**DOI:** 10.1007/s00259-024-06815-6

**Published:** 2024-06-26

**Authors:** Carla Oliveira, Francisco Oliveira, Cláudia Constantino, Celeste Alves, Maria José Brito, Fátima Cardoso, Durval C. Costa

**Affiliations:** 1 Nuclear Medicine-Radiopharmacology, Champalimaud Clinical Centre/Champalimaud Foundation, Lisbon, Portugal; 2 Breast Unit, Champalimaud Clinical Centre/Champalimaud Foundation, Lisbon, Portugal; 3 Pathology Department, Champalimaud Clinical Centre/Champalimaud Foundation, Lisbon, Portugal

**Keywords:** Breast cancer, Neoadjuvant chemotherapy, [^18^F]FDG PET/CT, Treatment response prediction, First-order features, pCR prediction

## Abstract

**Purpose:**

To verify the ability of pretreatment [^18^F]FDG PET/CT and T1-weighed dynamic contrast-enhanced MRI to predict pathological complete response (pCR) after neoadjuvant chemotherapy (NAC) in breast cancer (BC) patients.

**Methods:**

This retrospective study includes patients with BC of no special type submitted to baseline [^18^F]FDG PET/CT, NAC and surgery. [^18^F]FDG PET-based features reflecting intensity and heterogeneity of tracer uptake were extracted from the primary BC and suspicious axillary lymph nodes (ALN), for comparative analysis related to NAC response (pCR vs. non-pCR). Multivariate logistic regression was performed for response prediction combining the breast tumor-extracted PET-based features and clinicopathological features. A subanalysis was performed in a patients’ subsample by adding breast tumor-extracted first-order MRI-based features to the multivariate logistic regression.

**Results:**

A total of 170 tumors from 168 patients were included. pCR was observed in 60/170 tumors (20/107 luminal B-like, 25/45 triple-negative and 15/18 HER2-enriched surrogate molecular subtypes). Higher intensity and higher heterogeneity of [^18^F]FDG uptake in the primary BC were associated with NAC response in HER2-negative tumors (immunohistochemistry score 0, 1 + or 2 + non-amplified by *in situ* hybridization). Also, higher intensity of tracer uptake was observed in ALN in the pCR group among HER2-negative tumors. No [^18^F]FDG PET-based features were associated with pCR in the other subgroup analyses. A subsample of 103 tumors was also submitted to extraction of MRI-based features. When combined with clinicopathological features, neither [^18^F]FDG PET nor MRI-based features had additional value for pCR prediction. The only significant predictors were estrogen receptor status, HER2 expression and grade.

**Conclusion:**

Pretreatment [^18^F]FDG PET-based features from primary BC and ALN are not associated with response to NAC, except in HER2-negative tumors. As compared with pathological features, no breast tumor-extracted PET or MRI-based feature improved response prediction.

**Supplementary Information:**

The online version contains supplementary material available at 10.1007/s00259-024-06815-6.

## Introduction

Breast cancer (BC) is the most common cancer type in women worldwide [1] and the leading cause of cancer death among women [2].

Neoadjuvant chemotherapy (NAC) is used for potentially surgically resectable BC, aimed at expanding the surgical indications and improving eligibility for breast-conserving surgery. A pathological complete response (pCR) after NAC in BC patients, defined as the absence of remaining invasive cancer in the breast and axillary lymph nodes (ALN) on pathological examination of the post-treatment surgical excision specimens (ypT0/Tis ypN0), has shown to correlate with long-term outcomes [3, 4]. However, less than half of the BC patients attain pCR after NAC, with better responses in human epidermal growth factor receptor 2 (HER2)-positive and triple-negative (TN) BC than in luminal HER2-negative tumors [5]. As the side effects of NAC can be significant, it is desirable to identify those patients with a higher likelihood of attaining pCR, to avoid unnecessary toxicities and costs [6, 7].

Magnetic resonance imaging (MRI) is the most accurate imaging modality for loco-regional tumor staging and evaluation of response to NAC in BC patients, the latter usually after a fixed number of chemotherapy cycles [8]. Recently, the predictive ability of pretreatment MRI for pCR after NAC has been under research. Most studies have been using features reflecting intratumor heterogeneity of contrast uptake on T1-weighted dynamic contrast-enhanced (DCE) MRI [9], on the assumption that the most aggressive tumors tend to have more irregular vascularization and angiogenesis [10].

^18^F-Fluorodeoxyglucose positron emission tomography/computed tomography ([^18^F]FDG PET/CT) is increasingly used in BC staging (from TNM stages IIB to IV) [11]. A higher [^18^F]FDG uptake in the primary BC was shown to correlate with more aggressive histopathological markers (e.g., higher tumor grade, negativity for estrogen receptors (ER) and TN status) [12, 13] and poorer survival [14]. Also, the decrease in [^18^F]FDG uptake in the breast tumor and ALN between pretreatment and interim [^18^F]FDG PET/CT was shown to predict pCR after NAC [15]. More recently, some authors reported an association between baseline [^18^F]FDG PET-based tumor uptake heterogeneity features and prediction of response to NAC [7]. However, consensual results are lacking, which may be due to the heterogeneity of methodologies and BC subtypes [7]. Moreover, most of the PET-based features reported as predictive of pCR after NAC are second-and/or higher-order ones [16–19], which lack robustness due to dependence on the different acquisition modes, reconstruction and post-processing parameters [20–22]. As first-order features do not contain information about the spatial intensity distribution, they are more stable and reliable than the second- and higher-order features when applied to PET imaging [22].

We aimed to evaluate the ability of baseline [^18^F]FDG PET/CT to predict pCR after NAC in BC patients. To increase the reproducibility of our results, we restrained the analysis to features reflecting the intensity and heterogeneity of [^18^F]FDG uptake (first-order features). A subanalysis was performed including first-order features extracted from baseline T1-weighted DCE MRI to evaluate their ability to predict pCR after NAC, when combined with the PET-based and clinicopathological features.

## Materials and methods

### Study design

This retrospective single-center study was conducted at the Champalimaud Clinical Centre/Champalimaud Foundation (Lisbon, Portugal) and approved by the Institutional Review Board and the Institutional Ethics Committee. Data from consecutive eligible BC patients who went from initial staging through surgery between May 2013 and December 2022 were retrieved from clinical files.

The inclusion criteria were as follows: female sex at birth; biopsy-proven no special type (NST) BC; [^18^F]FDG PET/CT examination for initial staging before starting chemotherapy (treatment naïve patients); NAC (after initial staging) followed by surgery. The exclusion criteria were histological type of BC other than NST and detection of distant disease spread at diagnosis.

The endpoint for this study was the prediction of pCR after NAC according to the American Joint Committee on Cancer System (AJCC), defined as the total disappearance of invasive cancer in all the tumor lesions (ypT0/Tis ypN0) on histopathological examination of the post-treatment surgical excision specimens. Whenever there was residual invasive cancer in the primary lesion and/or ALN, a non-pCR was considered.

### Clinical and pathological data

The following clinical and pathological characteristics were retrieved from the clinical files: patient age at diagnosis; menopausal status; clinical tumor (cTcN) stage; tumor grade (1–2 vs. 3); unifocal vs. multifocal/multicentric primary BC; Ki67 index; HER2 immunohistochemistry (IHC) score. The tumors were considered HER2-negative if the IHC score was 0, 1 + or 2 + non-amplified by in situ hybridization (ISH) and HER2-positive if the IHC score was 3 + or 2 + amplified by ISH [23]. The tumors were classified according to the molecular subtype using as surrogates ER/progesterone receptors (PgR)/HER2 status and Ki67 index. The surrogate molecular subtypes were classified as luminal B-like, when more than 10% of the tumor cells were immunoreactive for ER [24], with either low PR (a cutoff of 20% was considered [25]) or high Ki67 (a cutoff of 15% was considered) [26, 27], with or without HER2 positivity; HER2-enriched for tumors with both negative ER and PgR and HER2 positivity; and TN, when less than 10% of the cells expressed ER and both PgR and HER2 were negative [26–28]. No luminal A-like tumors were included, because patients with this surrogate molecular subtype did not undergo [^18^F]FDG PET/CT for staging and did not receive NAC.

The standard NAC scheme consisted of an anthracycline and/or taxane and/or platinum regimen (plus anti-HER2 agents in patients with HER2-positive tumors by ISH or with IHC score 3+), as decided in a multidisciplinary tumor board.

NAC was followed by breast-conserving surgery or mastectomy with sentinel lymph node biopsy and/or ALN dissection.

Surgical specimens were analyzed by a pathologist with more than 10 years of experience using standard procedures for post-operative tissue to determine the response to NAC.

### [^18^F]FDG PET/CT image acquisition

For [^18^F]FDG PET/CT imaging, patients fasted for at least 4 h (blood glucose level was confirmed to be < 200 mg/dl) before the [^18^F]FDG injection (3.48 ± 0.29 MBq/kg of patient weight). The images were acquired approximately 60 min later, on a Philips Gemini TF 16 (Time of Flight) or Philips Vereos Digital PET/CT scanner, with the patient in dorsal decubitus. The imaging protocol included a low-dose CT (120 kV, 60 mA per rotation) from the skull base to the upper third of the thighs, followed by [^18^F]FDG emission data with a sequence of 7 to 11-bed positions (70 s per axial field of view, matrix 144 × 144). All [^18^F]FDG PET images were corrected for attenuation using the acquired CT data. All [^18^F]FDG PET images were acquired and reconstructed using protocols that fulfill EARL F-18 standards 1 specifications from the European Association of Nuclear Medicine Research Ltd [29].

### [^18^F]FDG PET/CT image analysis

The primary BC and (when applicable) the suspicious ipsilateral ALN were volumetrically identified on [^18^F]FDG PET images using 3D Slicer 4.11.20210226 [30]. A semiautomatic segmentation algorithm based on a Bayesian classifier, previously developed and validated in this type of data [31] was applied to all these lesions on PET images. The segmentation was performed by a nuclear medicine physician with more than 10 years of experience. In multifocal/multicentric BC, the largest tumor lesion was selected for analysis; if contiguous satellite lesions were found, they were also included along with the dominant lesion.

Only previously proven reproducible metabolic characteristics in the two available PET/CT systems were utilized [20]. First-order features/variables were extracted from the segmented primary tumor and ALN. Intensity-based features were measured based on the standardized uptake value (SUV) scale. The complete list of [^18^F]FDG PET-based extracted features was: energy [32], entropy [32], kurtosis [32], maximum SUV (SUV_max_), mean SUV (SUV_mean_), median SUV (SUV_median_), peak SUV (SUV_peak_), range [32], skewness [32], standard deviation (SD) [32], uniformity [32], coefficient of variation (CoV), metabolic tumor volume (MTV) and total lesion glycolysis (TLG).

### Statistical analysis

#### Univariate statistical analysis

All the quantitative breast tumor- and ALN-based [^18^F]FDG PET features were compared according to the NAC response (pCR vs. non-pCR) using the Mann-Whitney U test. The comparison was performed for: (a) the whole patient sample; (b) the same surrogate molecular subtype; and (c) the same HER2 status, whatever the surrogate molecular subtype (where a binary classification was considered for statistical purposes as: negative for IHC score 0, 1+, or 2 + with ISH negative vs. positive for IHC score 2 + with ISH positive or score 3+). The HER2-negative subgroup included luminal B-like HER2-negative and TN tumors. The HER2-positive subgroup included luminal B-like HER2-positive and HER2-enriched tumors.

For each clinical, pathological and [^18^F]FDG PET-based feature of the primary BC (making a total of 37 features), a receiver operating characteristic (ROC) analysis was performed to evaluate the respective discriminatory ability to correctly assign the patient into a two-group classification (i.e., pCR vs. non-pCR).

#### Multivariate statistical analysis

Before performing multivariate analysis, a feature selection process was defined using the following criteria: (a) only features with statistically significant predictive value for NAC response, based on the area under the ROC curve (AUC), were included; (b) whenever highly correlated features were found (absolute value of the Spearman’s correlation coefficient higher than 0.85), the ones with lower AUC for NAC response prediction were excluded from further analysis.

The selected features were used for multivariate logistic regression to predict pCR. For the selection of the features inside the Logistic Regression module, the “backward conditional method” was chosen. The adequacy of the final multivariable model was evaluated through the determination of the AUC interval, Nagelkerke R square and significance of the Omnibus test.

All the statistical analyses were performed with SPSS software (SPSS Inc., Chicago. IL, USA) version 20. A two-sided *p*-value below 0.05 was considered statistically significant.

### Subanalysis combining [^18^F]FDG PET and MRI-based features

Only those patients with the pretreatment MRI study performed in our Institution were included in this subanalysis, to ensure the images were acquired with the same equipment [Philips Magnetic Resonance Ingenia 3.0T - dStream broadband technology (The Netherlands)] and under the same study protocol. One-minute post-gadolinium injection images from the dynamic sequence T1 high-resolution isotropic volume excitation (with TR/TE = 3.2/1.55 ms and flip angle 12º) were used for segmentation of the primary BC with the same methodology as applied for [^18^F]FDG PET image segmentation. MRI-based lesion segmentation was verified by a breast-dedicated radiologist with more than 10 years of experience. Since MRI signal intensity is a combination of tissue properties and hardware-specific settings [33, 34], intensity-normalized versions of the maximum, mean and median signal were computed by dividing their value by the median signal of the corresponding non-tumoral contralateral breast tissue. The complete list of the analyzed MRI-based features (which were measured based on signal intensity) was: energy [32], entropy [32], kurtosis [32], range [32], skewness [32], SD [32], uniformity [32], CoV, tumor volume, normalized maximum (N_max), normalized mean (N_mean) and normalized median (N_median).

Univariate and multivariate analyses were performed in this subsample according to the same criteria that were used for the main sample.

## Results

### Patient characteristics and pathological outcomes

Demographic and clinical characteristics of all the included patients are summarized in Table 1. A total of 168 patients (170 tumors) were included. In 123 tumors (72%) there was ALN involvement. Overall, pCR was observed in 60 tumors (35%) and did not occur in 110 (65%).

**Table 1:** Patients and tumors’ characteristics

Patients’ characteristics (*n* = 168)	
**Age**	**Mean (SD)** **52(11)**
**Menopausal status** ** Pre-menopausal** **Post-menopausal** *Missing data*	***n*** 80 87 1
**Tumors’ characteristics (** ***n*** ** = 170)**	
	**n (%)**
**Clinical T stage (cT)**	
***cT1*** ***cT2*** ***cT3*** ***cT4***	29 (17%) 87 (51%) 35 (21%) 19 (11%)
**Clinical N stage (cN)**	
***cN0*** ***cN+***	47 (28%) 123 (72%)
**Unifocality of the primary tumor**	
***Yes*** ***No*** *Missing data*	99 (58%) 67 (39%) 4
**Estrogen receptors (≥ 1%)**	
**Positive** **Negative**	107 (63%) 63 (37%)
**Progesterone receptors (≥ 1%)**	
**Positive** **Negative**	95 (56%) 75 (44%)
**Grade**	
**1–2** **3** *Missing data*	80 (47%) 88 (52%) 2
**Ki67** **Mean (SD)**	**(%)** 53.14 (21.90)
**NAC response**	
**pCR** **non-pCR**	**n (%)** 60 (35%) 110 (65%)

SD - standard deviation

The distribution of patients with pCR vs. non-pCR in each surrogate molecular subtype as well as according to HER2 expression is presented in Table 2. Detailed information about the distribution of tumors with ALN involvement is given in Supplementary Table 1.

**Table 2:** NAC response according to the tumor surrogate molecular subtypes and HER2 expression

Tumors’ characteristics	Total tumor sample		
	pCR *n* (%)	Non-pCR *n* (%)	Total (*n*)
Luminal B-like	20 (19%)	87 (81%)	**107**
TN	25 (56%)	20 (44%)	**45**
HER2-enriched	15 (83%)	3 (17%)	**18**
HER2 negativity	27 (21%)	99 (79%)	**126**
HER2 positivity	33 (75%)	11 (25%)	**44**
**Total (n)**	**60**	**110**	**170**

### Comparison between pCR and non-pCR groups

The median, 1st and 3rd quartiles (Q1-Q3) for the [^18^F]FDG PET-based features in the function of the NAC response are shown in Table 3. In the total tumor sample, the PET-based CoV was significantly higher in the pCR than in the non-pCR group. In HER2-negative BC, the following [^18^F]FDG PET-based features were significantly different between the pCR and non-pCR tumors: uniformity (lower in patients with pCR); entropy, SUV_max_, SUV_mean_, SUV_median_, range, SD and CoV (higher in patients with pCR). There was no significant association of any analyzed [^18^F]FDG PET-based feature with response to NAC among luminal B-like or TN subtypes nor among HER2-positive tumors.

**Table 3:** Significant PET features in the primary BC in the prediction of pCR on Mann-Whitney U test

Tumor characteristics	PET features (*p* < 0.05)	Median (Q1-Q3)	
		pCR	Non-pCR
Total (*n* = 170)	CoV	0.36(0.24–0.45)	0.30(0.17–0.40)
Luminal B-like (*n* = 107)	*None*		
TN (*n* = 45)	*None*		
HER2-enriched (*n* = 18)	*Not analyzed (small sample)*		
HER2 negativity (*n* = 126)	Entropy SUV_max_ SUV_mean_ SUV_median_ Range SD Uniformity CoV	3.18(2.38–3.62) 13.04(7.21–18.98) 6.29(4.08–8.21) 4.84(3.79–6.92) 9.60(4.58–15.19) 2.45(1.10–3.58) 0.05(0.03–0.10) 0.38(0.30–0.50)	2.63(1.72–3.33) 7.82(4.53–15.70) 4.42(3.00-6.94) 4.06(2.90–6.19) 4.94(1.89–11.51) 1.21(0.45–2.77) 0.09(0.04–0.20) 0.29(0.16–0.40)
HER2 positivity (*n* = 44)	*None*		

In HER2-negative tumors, from the analyzed [^18^F]FDG PET-based features in ALN (Table 4), SUV_max_, SUV_mean_ and SUV_median_ were higher in the pCR group than in the non-pCR group. In the ALN of luminal B-like and TN subtypes and HER2-positive BC, no significant difference between responders and non-responders was found in any of the analyzed PET-based features.

**Table 4:** Significant PET features in ALN in the prediction of pCR on Mann-Whitney U test

Tumor characteristics	PET features (*p* < 0.05)	Median (Q1-Q3)	
		pCR	Non-pCR
Total (*n* = 123)	*None*		
Luminal B-like (*n* = 83)	*None*		
TN (*n* = 27)	*None*		
HER2-enriched (*n* = 13)	*Not analyzed (small sample)*		
HER2 negativity (*n* = 94)	SUV_max_ SUV_mean_ SUV_median_	8.22(5.64–17.36) 4.59(3.62–6.51) 4.56(3.37–5.96)	5.19(3.54–10.51) 2.96(2.23–4.66) 2.92(2.18–4.30)
HER2 positivity (*n* = 29)	*None*		

**Fig. 1:**
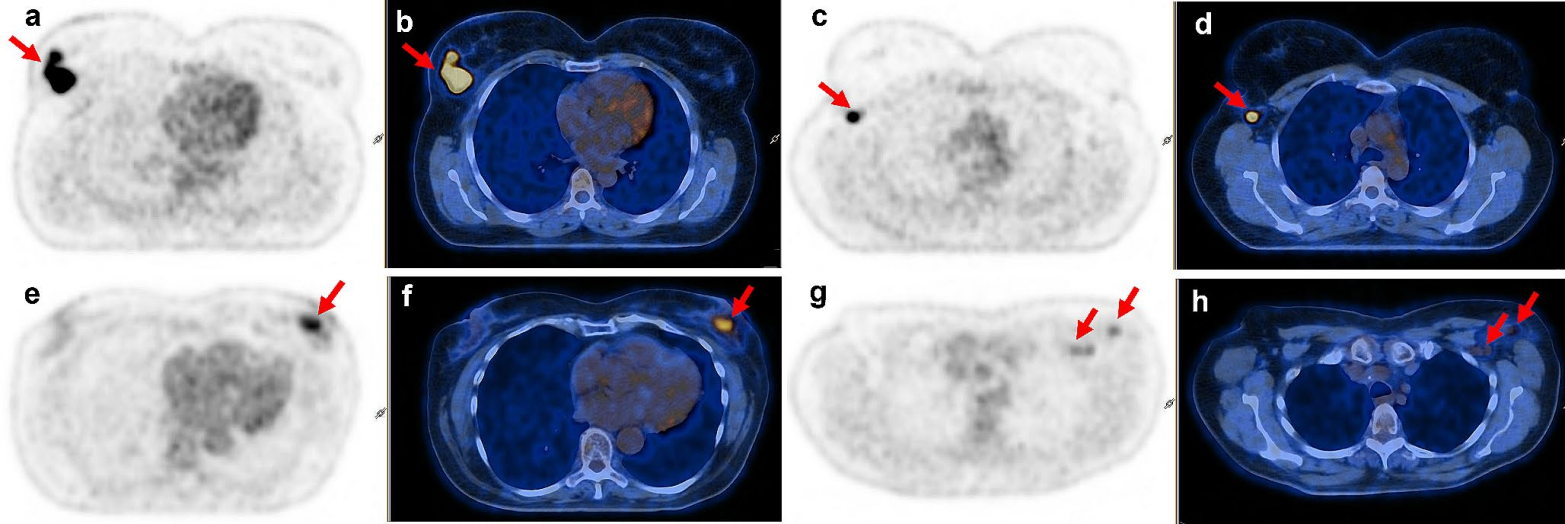
Pretreatment [^18^F]FDG PET only and fusion PET/CT images of two patients with HER2-negative BC and ipsilateral ALN involvement. **a**, **b**, **c**, **d**: TN BC with pCR after NAC. **a**, **b**: Primary tumor (SUV_mean_=8.21; CoV = 0.54); **c**, **d**: ALN (SUV_mean_=3.30); **e**, **f**, **g**, **h**: Luminal-B like BC with non-pCR after NAC; **e**, **f**: Primary tumor (SUV_mean_=3.32; CoV = 0.17); **g**, **h**: ALN (SUV_mean_=2.22)

Representative PET/CT images of patients with HER2-negative tumors exhibiting pCR and non-pCR after NAC are shown in Fig. 1.

### Predictive model building

From the features included in the univariate analysis of the whole sample (Table 5), only ER and HER2 expression were selected for the multivariate analysis final predictive model, with a 95% confidence AUC interval of [0.824; 0.929].

**Table 5:** Results from feature selection and multivariable logistic regression predicting pCR after NAC in the whole tumor sample

Characteristics	Univariate analysis		Multivariate analysis					
	Clinical and pathological features (AUC interval > 0.5)	PET features (AUC interval > 0.5)	Selected features from univariate analysis	Features in the final model	Odds Ratio (95% CI)	AUC interval	Nagelkerke *R* square	*p* (Omnibus test)
Total (*n* = 167)^a^	Grade ER^c^ PgR^c^ HER2 Ki67	Range^d^ CoV^d^	Grade ER HER2 Ki67 CoV	ER^f^ HER2^g^	0.06(0.02–0.17) 26.3(8.6–80.2)	[0.824;0.929]	0.532	< 0.001
Luminal B-like (*n* = 107)	HER2	*None*	*Not performed*					
TN (*n* = 45)	*None*	*None*	*Not performed*					
HER2-enriched (*n* = 18)	*Not performed (small sample)*							
HER2 negativity (*n* = 123)^b^	Clinical T stage (cT) Grade ER^c^ PgR^c^ Ki67	Entropy^d, e^ SUV_max_^d, e^ SUV_mean_^d^ SUV_median_^d^ Range^d, e^ SD^d, e^ Uniformity^d, e^ CoV^e^	cT Grade ER Ki67 SUVmean CoV	ER^f^	0.02(0.01–0.10)	[0.787;0.937]	0.499	< 0.001
HER2 positivity (*n* = 44)	*None*	*None*	*Not performed*					

^a^Three tumors were excluded (2 due to missing grade and 1 due to missing Ki67)

^b^Three tumors were excluded (2 due to missing grade and 1 due to missing Ki67)

^c, d,e^Correlated features (Spearman coefficient higher than 0.85)

^f^Reference is ER-negative

^g^Reference is HER2-negative

In HER2-negative tumors, ER was the only feature selected for the multivariate analysis final model, with a 95% confidence AUC interval of [0.787; 0.937].

Among luminal B-like tumors, only HER2 status showed an AUC above 0.5 in the ROC analysis for predicting pCR, so further analysis was not performed, since our focus was to verify if PET-based features could be included in the predictive model. In TN and HER2-positive tumors, no clinical or pathological nor any PET-based feature seemed to be predictive of pCR (AUC interval not significant), so multivariate logistic regression was also not performed in these subgroups. Due to the small sample size, multivariate logistic regression was not performed in the HER2-enriched BC subgroup.

### Subanalysis combining [^18^F]FDG PET and MRI-based features

To evaluate the ability of MRI-based features to predict pCR when combined with PET-based and clinicopathological features, a subanalysis was performed in 102 patients (103 tumors: 64 luminal B-like, 30 TN and 9 HER2-enriched subtypes). ALN involvement was present in 77 (75%) of the tumors. Overall, pCR was observed in 36 (35%) and did not occur in 67 (65%) tumors.

The distribution of patients with pCR vs. non-pCR in each surrogate molecular subtype as well as according to HER2 expression is presented in Supplementary Table 2.

In the predictive model building for pCR (Table 6), grade, ER, HER2 expression and MRI-based kurtosis (which describes the flatness of the peak of the distribution curve of signal intensity) were selected for the multivariate analysis final model of the whole subsample, with a 95% confidence AUC interval of [0.875; 0.971]. Although there was a trend, in the final model, relating kurtosis (MRI-based) to pCR prediction, it was not significant (*p* = 0.058).

**Table 6:** Results from feature selection and multivariable logistic regression predicting pCR after NAC in the subsample with analyzed MRI

Characteristics	Univariate analysis	Multivariate analysis					
	MRI features (AUC interval > 0.5)	Selected features from univariate analysis	Features in the final model	Odds Ratio (95% CI)	AUC interval	Nagelkerke *R* square	*p* (Omnibus test)
Total (*n* = 102)^a^	Kurtosis	Grade ER HER2 Ki67 CoV (PET) Kurtosis (MRI)	Grade^d^ ER^e^ HER2^f^ Kurtosis (MRI)	0.14(0.02–0.80) 0.02(0.002–0.21) 137(13-1441) Not significant	[0.875;0.971]	0.687	< 0.001
Luminal B-like (*n* = 64)	Kurtosis N_Mean^c^ N_Median^c^	HER2 Kurtosis (MRI) N_Mean (MRI)	HER2^f^	Not significant	[0.820–9.88]	0.800	< 0.001
TN (*n* = 30)	*None*	*Not performed*					
HER2-enriched (*n* = 9)	*Not performed (small sample)*						
HER2 negativity (*n* = 77)^b^	*None*	*Not performed*					
HER2 positivity(luminal B-like HER2-positive and HER-enriched tumors) (*n* = 25)	*Not performed (small sample)*						

^a^One tumor was excluded due to missing grade.

^b^One tumor was excluded due to missing Ki67.

^c^Correlated features.

^d^Reference value is grade 3.

^e^Reference value is ER-negative.

^f^Reference value is HER2-negative

Among luminal B-like tumors, HER2 expression was the only feature selected for the multivariate analysis final model, but it was not a significant predictor (*p* = 0.997).

As no MRI-based feature seemed to predict pCR (AUC interval not significant) in the TN and HER2-negative subgroups, multivariate logistic regression was not performed in these subgroups. Due to the small sample size, multivariate logistic regression was not performed in the HER2-enriched nor in the HER-positive subgroups.

## Discussion

In the present study, first-order PET-based features were not predictive of pCR in BC, except in HER2-negative tumors. In this BC subtype, higher [^18^F]FDG uptake (higher SUV_max_, SUV_mean,_ and SUV_median_) and higher [^18^F]FDG uptake heterogeneity of distribution (higher entropy, range, SD and CoV and lower uniformity) in the primary BC were associated with pCR after NAC. Higher [^18^F]FDG uptake (higher SUV_max_, SUV_mean_ and SUV_median_) in the ALN was also associated with pCR after NAC in HER2-negative BC.

Our findings are not in line with the thesis that BC with higher heterogeneity of tracer uptake on baseline [^18^F]FDG PET/CT have a worse response to NAC [35]. The predictive parameters found in Yoon HJ et al [35] were texture based-PET features that we did not analyze [35]. Also, the different pathologic endpoints for the assignment of tumors into the responder group (AJCC system with the endpoint of pCR in our study vs. Sataloff system with partial or complete response in the other study [35]) may also account for the non-consistency of the results [35]. As pCR after NAC in BC has been considered a surrogate of long-term outcomes [3, 4], our results do not support the thesis that breast tumors with higher metabolic heterogeneity on baseline [^18^F]FDG PET/CT have worse long-term outcomes [36]. However, the different sample characteristics (a larger sample of TN BC [36] than the one collected in our study) and comparison of different outcomes make these studies not directly comparable. Moreover, it should be emphasized that we focused only on PET-based features reflecting glycolytic metabolism, namely intensity and heterogeneity of [^18^F]FDG uptake. These analyzed PET-based features represent a tiny part of the intratumor heterogeneity in BC, that encompasses a panoply of genetic, phenotypic and microenvironmental heterogeneous features. This complexity of the factors makes it impossible to evaluate the whole burden of heterogeneity with a single radiopharmaceutical [37]. In addition, the spatial resolution of PET imaging does not allow assessing [^18^F]FDG consumption differences between regions closer than 4 to 6 mm [7].

Higher intensity and higher heterogeneity of [^18^F]FDG uptake have been reported for TN BC in comparison with non-TN BC [12]. Thus, since the majority of the analyzed PET-based features that were associated with response to NAC occurred among BC HER2-negative tumors, our findings can reflect the known higher probability of TN BC to achieve pCR in comparison with luminal B-like subtype with no HER2 amplification [38]. Within the TN BC subgroup, no significant differences were observed for the PET-based features between the pCR and non-pCR groups.

Other authors [35, 39, 40] did not find any significant association between [^18^F]FDG PET-based first-order features in the primary BC and response to NAC, using tests for two independent samples. Li et al. [18] also did not consider any first-order PET-based feature as predictive of pCR, using machine learning models. In the work of Ha et al. [19], an association with NAC was obtained for a set of PET-based parameters containing first-order features, namely CoV and skewness. Roy et al. [41] included PET-based QRobust mean absolute deviation in the top four features that constituted the radiomic signature used for pCR prediction in TN BC. Both studies [19, 41] used machine learning models and included PET-based texture features along with the first-order ones in the resulting tumor clusters [19] and in the radiomic signature for prediction [41].

As to the MRI-based features, Granzier et al. [42] did not find additional value of signal intensity and/or heterogeneity-reflecting first-order features extracted from the primary BC on baseline T1-weighted DCE MRI to the clinical models predictive of pCR. However, other authors included first-order features in the final predictive model (along with texture-based features), namely: kurtosis and 10th percentile in the study of Peng et al [43]; range in the study of Pesapane et al. [44]; variance, entropy, and the 90th percentile in the study of Bitencourt et al. [45].

With the growing availability of integrated PET/MRI systems, further exploitation of the pCR prediction can take advantage of the spatial and temporal correlation of the simultaneously acquired PET and MRI data [46], optimizing the reproducibility of the results. Also, both modalities may benefit from automated and accurate co-registration in the delineation of the tumors.

In our study, none of the analyzed PET-based features added statistically significant value for pCR prediction compared to pathological factors. The only predictive features in multivariate analysis were ER and HER2 expression in the total sample and ER status in HER2-negative tumors. These findings are in agreement with the known higher probability of tumors with negative ER and HER2 positivity to achieve pCR after NAC [38]. In the subanalysis with [^18^F]FDG PET and MRI-based features, MRI-based kurtosis was included in the final model for the whole subsample, however, it was not significant in the final model. The only significant predictive factors for pCR in this subanalysis were ER, HER2 and grade in the total subsample, the latter reflecting the higher probability of higher-grade BC patients achieving pCR after NAC [47]. 

Our study has some limitations. First, the relatively small sample size and its heterogeneity, due to the diversity of BC subtypes. Also, the proper heterogeneity within each surrogate molecular subtype, meaning that distinct BC molecular subtypes can have phenotypic variance in an apparently uniform tumor, which complicates our analysis and can introduce biases in the prediction models [48]. In addition, the subanalysis combining PET and MRI-based features was performed in only a subsample of tumors, from patients evaluated at our institution, with the same MRI equipment. However, we emphasize the relevance of our results, that were based on a limited set of first-order [^18^F]FDG uptake-related features for pCR prediction, compared to most previous studies that have used a wide range of texture-based features, lacking robustness and promoting statistical associations by chance. Moreover, few studies have analyzed the association between PET-based features of ALN and response to NAC [7].

## Conclusion

In our study, baseline [^18^F]FDG PET-based first-order features were not associated with pCR after NAC in most NST BC subtypes, except in HER2-negative tumors. In the multivariate analysis final model, none of the PET or MRI-based features was relevant for pCR prediction. ER and HER2 status were the main determiners of pCR in the whole sample and in the subgroup analyses.

## Electronic supplementary material

Below is the link to the electronic supplementary material.

Supplementary Material 1

## Data Availability

The datasets generated and analysed during the current study are available from the corresponding author upon reasonable request.
