# Supplementary material for: Baseline [18F]FDG PET/CT and MRI first-order breast tumor features do not improve pathological complete response prediction to neoadjuvant chemotherapy
Source: Eur J Nucl Med Mol Imaging. 2024 Jun 26;51(12):3709–18. doi: 10.1007/s00259-024-06815-6 (PMC11445295; doi:10.1007/s00259-024-06815-6)
Supplement: Supplementary file 1 — Supplementary Material 1 [file 259_2024_6815_MOESM1_ESM.docx]

***European Journal of Nuclear Medicine and Molecular Imaging***

***Baseline [^18^F]FDG PET/CT and MRI first-order breast tumor features do not improve pathological complete response prediction to neoadjuvant chemotherapy***

Carla Oliveira^1^, Francisco Oliveira^1^, Cláudia Constantino^1^, Celeste Alves^2^, Maria José Brito^2,3^, Fátima Cardoso^2^, Durval C. Costa^1^

*^1^Nuclear Medicine-Radiopharmacology, Champalimaud Clinical Centre/Champalimaud Foundation, Lisbon, Portugal*

*^2^Breast Unit, Champalimaud Clinical Centre/Champalimaud Foundation, Lisbon, Portugal*

*^3^Pathology Department, Champalimaud Clinical Centre/Champalimaud Foundation, Lisbon, Portugal*

Corresponding author:

Carla Oliveira

Email: [carla.oliveira@fundacaochampalimaud.pt](mailto:carla.oliveira@fundacaochampalimaud.pt)

**Supplementary Table 1 – Neoadjuvant chemotherapy response in tumors from patients with axillary lymph node involvement (ALN) according to the tumor surrogate molecular subtypes and HER2 expression**

| **Tumors’ characteristics** | **Tumors from patients with involved ALN** | | |
| --- | --- | --- | --- |
|  | **pCR**  **n (%)** | **Non-pCR**  **n (%)** | **Total (n)** |
| Luminal B-like | 14 (17%) | 69 (83%) | **83** |
| TN | 15 (56%) | 12 (44%) | **27** |
| HER2-enriched | 12 (92%) | 1 (8%) | **13** |
| HER2 negativity | 17 (18%) | 77 (82%) | **94** |
| HER2 positivity | 24 (83%) | 5 (17%) | **29** |
| **Total (n)** | **41** | **82** | **123** |

pCR – Pathological complete response

**Supplementary Table 2 –** **Neoadjuvant chemotherapy response according to the tumor surrogate molecular subtype and HER2 expression in the subanalysis with [^18^F]FDG PET and MRI-based variables**

| **Tumors’ characteristics** | **Total tumor subsample** | | |
| --- | --- | --- | --- |
|  | **pCR**  **n (%)** | **Non-pCR**  **n (%)** | **Total (n)** |
| Luminal B-like | 12 (19%) | 52 (81%) | **64** |
| TNBC | 16 (53%) | 14 (47%) | **30** |
| HER2-enriched | 8 (89%) | 1 (11%) | **9** |
| HER2 negativity | 16 (21%) | 62 (79%) | **78** |
| HER2 positivity | 20 (80%) | 5 (20%) | **25** |
| **Total (n)** | **36** | **67** | **103** |

pCR – Pathological complete response
